# Use of adenosine deaminase (ADA) to diagnose suspected peritoneal tuberculosis in Rwanda: a cross-sectional study

**DOI:** 10.1186/s12879-020-04965-0

**Published:** 2020-03-20

**Authors:** Jules Ntwari, Vincent Dusabejambo, Cameron Page

**Affiliations:** 1 University of Rwanda, College of Medicine and health sciences, Kigali, Rwanda; 2 University Hospital of Brooklyn, SUNY Downstate Medical Center, Brooklyn, NY USA

**Keywords:** Tuberculosis, Rwanda, Infectious disease, Adenosine deaminase

## Abstract

**Background:**

Peritoneal tuberculosis is the most common cause of low albumin gradient ascites in developing countries, but it can be easily confused with other causes of ascites. Peritoneal tuberculosis requires early recognition of symptoms and signs in order to make a quick diagnosis for appropriate treatment**.** Measurement of adenosine deaminase (ADA) level > 39 in ascites fluid is an established test to diagnose peritoneal tuberculosis. Many low-income countries do not currently test for adenosine deaminase in ascites fluid, including Rwanda.

**Method:**

Cross-sectional, descriptive study conducted through the Internal Medicine Department of three university teaching hospitals in Rwanda. Participants were patients older than 16 years presenting to tertiary referral hospitals with ascites of unknown cause.

**Results:**

Of 103 ascites fluid samples collected, 52 of them (50.5%) had an elevated ADA, consistent with a presumptive diagnosis of peritoneal TB. Among those 52 subjects diagnosed with peritoneal TB, 39 out of 52 (75%) did not receive anti-TB medications. Among the 17 subjects who were treated with anti-TB medications, 4 of 17 (23.6%) did not have peritoneal TB based on ADA level. Samples with low-albumin gradient ascites were more likely to have high ADA ≥39 IU/L (*p* = 0.039).

**Conclusion:**

Our findings suggest that 3out of 4 patients with PTB in Rwanda are not getting TB treatment and 1 in 4 patients who are taking TB medications do not need it. Even if the true number of Rwandans who are being undertreated and overtreated is less than our study suggests, these results should prompt a larger study of peritoneal tuberculosis. Adding adenosine deaminase (ADA) to the diagnostic tools available to clinicians could help achieve the goal of correctly putting every Rwandan with tuberculosis on treatment, while avoiding unnecessary tuberculosis medications in those who do not have the disease.

## Background

Tuberculosis (TB) remains a major global health problem. It leads to disease for approximately 10 million people every year and it is among the top ten causes of death worldwide. Worldwide, prevalence of peritoneal tuberculosis represents 5% of patients with *Mycobacterium tuberculosis* infection [1, 2].

Nearly 6000 cases of tuberculosis were reported in Rwanda in 2015, and 84% of them were pulmonary TB. According to the Global Tuberculosis Report of 2017, tuberculosis incidence in Rwanda was 56/100,000 people and the mortality rate was 3.8/100,000 [2, 3].

The diagnosis of peritoneal tuberculosis is challenging because it is hard to differentiate from other intra-abdominal diseases that produce ascites. Furthermore, the slow growth of mycobacterial cultures means that bacteriologic confirmation is impractical, in a setting where many patients are unable to follow up for culture results after they leave the hospital [4–6].

Measurement of adenosine deaminase (ADA) in ascites fluid has been studied as a useful non-culture method of detecting peritoneal tuberculosis. A recent meta-analysis of 12 prospective studies, including 1201 subjects, found that ADA levels had high sensitivity (100%) and specificity (97%) by using cut-off values between 36 and 40 IU/L; the optimal cut-off value was 39 IU/L [4, 7].

There are several reasons why a rapid minimally-invasive test such as ADA to diagnose peritoneal tuberculosis is useful. First, it can prevent the treatment delay that occurs while waiting for slow-growing culture results. Second, it avoids the waste of resources caused by giving anti-tuberculosis drugs to patients who don’t have TB. Third, it reduces the unnecessary hepatotoxicity associated with giving anti-TB drugs to patients who do not have tuberculosis. Fourth, the use of ADA can help avoid the need for risky invasive techniques such as laparoscopic peritoneal biopsy [4, 8].

The purpose of this study was to determine whether adding ADA to the current diagnostic tools in Rwanda would assist in giving patients with ascites the correct treatment. Our goal is to determine if ADA can help to put every patient with peritoneal tuberculosis on anti-TB medications, and also avoid giving unnecessary TB medications to patients who do not have tuberculosis.

## Methods

### General objective

To determine the utility of adenosine deaminase (ADA) as diagnostic tool for peritoneal tuberculosis at referral hospitals in Rwanda.

### Specific objectives

1. To determine the prevalence of peritoneal TB diagnosed by ADA in patients with ascites.
2. To determine whether ADA correlates with other noninvasive ascites fluid analysis tests and if ADA is correlated with any clinical characteristic.

### Study design

Cross-sectional, descriptive.

### Study setting

The study was conducted from January to December 2017 at Kigali University Teaching Hospital (KUTH), Butare University Teaching Hospital (BUTH) and Rwanda Military Hospital (RMH). These are the main public health institutions and referral hospitals in Rwanda. KUTH and BUTH are referral and university teaching hospitals. KUTH has 429 beds and is located in the center of Kigali City. It receives referred patients from all district hospitals. RMH is a military hospital that is also located in Kigali, 17 km from KUTH. The third hospital is BUTH, which has 418 beds and is located in the Southern province approximately 125 km from the capital city of Kigali.

### Study population

The study population was patients who presented to one of the above-mentioned tertiary referral hospitals with new clinical features of ascites at admission. Clinical features used to determine presence of ascites included abdominal distension, bulging flanks, and shifting dullness. If available, ultrasound was used to confirm the presence of ascites. Patients were recruited in this study through the Emergency Department or the Internal Medicine department. The decision to perform a paracentesis was made by the treating physician, not the researcher.

### Inclusion criteria

- 16 years and above

Patient presenting with newly diagnosed unknown cause of ascites.

### Exclusion criteria

- Patients with a known diagnostic etiology of ascites who presented for therapeutic paracentesis

### Data collection and analysis

After the diagnosis of ascites was made, eligible subjects were explained about the study. Ascites fluid samples were collected prospectively by diagnostic paracentesis procedure in aseptic condition and sample was sent immediately to the laboratory at the respective hospital (KUTH, BUTH or RMH) where the subject was located. Ascites fluid samples were stored in the laboratory freezer which was maintained at minus eighty Celsius degree (**−** 80 C) until day of ADA testing.

Clinical and socio-demographic information was obtained from each subject by verbal interview. Laboratory and imaging data were obtained though medical or electronic record. Each ascites sample was recorded in a register which was kept at the laboratory by technician, and a second register was kept by the investigator containing the subject’s registration number. Hepatitis B or C cirrhosis were diagnosed using a combination of impaired liver function tests, presence of HBsAg or Anti-HCV Ab, clinical evidence of portal hypertension, and abnormal imaging techniques (e.g. ultrasound, CT scan). Diagnosis of alcoholic cirrhosis was made by combination of impaired liver function tests, evidence of portal hypertension, abnormal imaging findings and history of significant alcohol consumption. Nonalcoholic fatty liver disease was diagnosed by presence of hepatic steatosis by imaging after exclusion of other causes of steatosis and no history of significant of alcohol consumption.

Congestive heart failure was diagnosed when the following criteria were met: cardiomegaly, radiological evidence of congested lungs, dyspnea, and peripheral edema. Renal failure was diagnosed when there was a raised urea and creatinine level in the presence of clinical evidence of fluid overload (e.g. pulmonary or peripheral edema), time course of disease and abnormal imaging findings of kidneys. The diagnosis of lymphoma, gastric adenocarcinoma and ovarian cancer was made by confirmation of tissue biopsy.

To test adenosine deaminase values in our study, we used the Adenosine Deaminase test kit DZ117-A-K Dual Vial Liquid Stable Format (Diazyme Corporation, Poway, CA, USA). The kit was stored at 2 to 8 degrees C until used. Ascites fluid samples were stored at minus eighty Celsius degree (− 80 C) until the day of analysis.

Sample analysis was done at KUTH by a trained lab technician and the investigator. On the day prior to ADA testing, samples collected at RMH and BUTH were transported to KUTH under frozen conditions at negative four degrees Celsius (− 4 C). After analysis the results were recorded in the register and in the data collection questionnaire.

### Statistical analysis

We used well-established criteria based on many prior studies for presumptively diagnosing subjects with peritoneal tuberculosis based on ADA [4, 9–11]. Subjects were divided into two categories: those with ADA ≥39 UI were classified as high ADA and considered to have peritoneal TB. Those with ADA < 39 were classified as low ADA and considered not to have peritoneal TB. Using these criteria, correlations were performed between peritoneal TB classification (positive or negative) and every demographic factor. For continuous variables such as age we used the student’s t-test (independent sample t test), and for categorical variables we used the Chi Square test.

Data analysis was done by using SPSS version 16.0. A *p*-value of < 0.05 was considered as evidence of a statistically significant difference.

### Data availability and ethical considerations

This study was approved by CMHS Institutional Review Board (IRB) of University of Rwanda, as well KUTH, BUTH and RMH ethical and research committees.

All participants in this study provided written consent to participate to the study, after explanation of the benefits of participation of this study. For patients who were very sick and unable to sign consent, the next of kin signed consent on their behalf. All personal health information provided by participant was kept in strict confidentiality.

The datasets used and analyzed during the current study are available from the corresponding author on reasonable request.

## Results

From January to December 2017, 103 subjects were enrolled, 47.6% of whom were female. The majority of patients were under 55 years old (76%) and the mean age was 44. Half of the subjects (49.5%) were from Kigali city, with the remainder from outlying districts (Table 1).

**Table 1:** Socio-demographic characteristics of subjects with newly diagnosed ascites at tertiary referral hospitals in Rwanda.

	Frequency (***n*** = 103)	Percentage (%)
**Age range**		
≤ 55 years	78	75.7
> 55 years	25	24.3
**Mean age**: 43.81 ± 16.79		
**Gender**		
Male	54	52.4
Female	49	47.6
**Residence**		
Kigali city	51	49.5
Eastern province	21	20.4
Northern province	6	5.8
Southern province	16	15.5
Western province	9	8.7

**Fig. 1:**
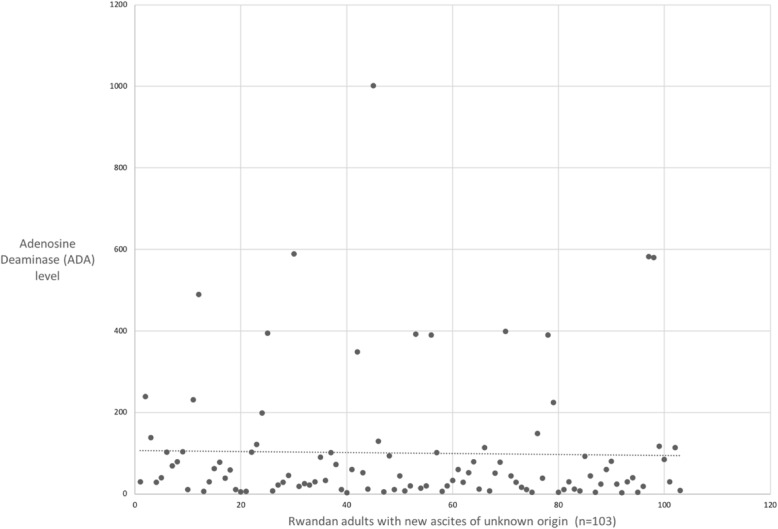
Adenosine Deaminase (ADA) levels in peritoneal fluid of patients presenting with new ascites to a tertiary referral hospital in Rwanda

Half of the cases of ascites in our study were from peritoneal tuberculosis (50.5%) (Table 2). There was a wide range of ADA values, from as low as 2 to above 1000, with an average ADA of approximately 100 (Fig. 1).

**Table 2:** Co-morbidities of patients presenting with new ascites to a tertiary referral hospital in Rwanda

	Frequency (***n*** = 103)	Percentage (%)
**CHF**	6	5.8
**Renal disease**		
Acute kidney injury	18	17.5
Chronic kidney disease	4	3.9
**Malignancy**		
Lymphoma	3	2.9
Gastric adenocarcinoma	1	1.0
Ovarian cancer	1	1.0
**Liver disease**		
Hepatitis B cirrhosis	19	18.4
Hepatitis C cirrhosis	13	12.6
Alcoholic cirrhosis	1	1.0
Nonalcoholic fatty liver disease	4	3.9
Idiopathic	6	5.8
**Diabetes mellitus**	4	3.9
**HIV**	12	11.7
No co-morbidity	11	10.7

We found that 43 subjects had cirrhosis (41.7%). Among these 43 subjects, viral hepatitis was the cause in 32 cases (74.4%), with hepatitis B (19 cases, 44.2%) more common that hepatitis C (13 cases; 30.2%). We found 22 cases of impaired renal function with elevated creatinine, and most of these (18/22) were acute kidney disease and resolved prior to discharge. The most common malignancy we found was lymphoma (3 / 5), and all of these cases were diagnosed in patients with HIV (Table 3).

**Table 3:** Co-morbidities of patients with new ascites at tertiary referral hospitals

	Frequency (***n*** = 103)	Percentage (%)
**CHF**	6	5.8
**Renal disease**		
Acute kidney injury	18	17.5
Chronic kidney disease	4	3.9
**Malignancy**		
Lymphoma	3	2.9
Gastric adenocarcinoma	1	1.0
Ovarian cancer	1	1.0
**Liver disease**		
Hepatitis B cirrhosis	19	18.4
Hepatitis C cirrhosis	13	12.6
Alcoholic cirrhosis	1	1.0
Nonalcoholic fatty liver disease	4	3.9
Idiopathic	6	5.8
**Diabetes mellitus**	4	3.9
**HIV**	12	11.7
No co-morbidity	11	10.7

The treating clinician, who was not aware of the ADA level, chose to give anti-TB medications in fewer than 1 in 5 cases (17 / 103; 16.5%). Among the 17 subjects who were treated for tuberculosis, 4 of them (23.5%) had low ADA level suggesting they did not have peritoneal TB. Of the 52 subjects who had a high ADA level ≥ 39 UI suggestive of tuberculosis, only 13 of them (25%) received anti TB treatment. Three out of four patients (75%) with peritoneal TB did not get treatment for their disease in our study (Table 4).

**Table 4:** Correlation between ADA value and peritoneal TB treatment

ADA range	Anti TB treatment		***P*** value
	Yes (***n*** = 17)	No (***n*** = 86)	
< 39 UI	4 (23.5%)	47 (54.7%)	0.019
≥39UI	13 (76.5%)	39 (45.3%)	

All subjects presented with abdominal distention, 70.9% had abdominal pain, 56.3% had weight loss, 30% had fever, and 26.2% had night sweats. We found no significant association between any presenting clinical features and their ADA levels. Subjects with abdominal pain and dyspnea had a slightly higher chance of having an elevated ADA than those without these features, but the differences between the groups were not statistically significant (Table 5).

**Table 5:** Correlation between clinical presenting features and ADA values

Clinical features (***N*** = 103)	ADA range		***P*** value
	< 39 UI	≥39 UI	
Abdominal pain	33 (45.2%)	40 (54.8%)	0.17
Abdominal distension	51 (49.5%)	52 (50.5%)	0.97
Fever	14 (45.2%)	17 (54.8%)	0.56
Cough	4 (33.3%)	8 (66.7%)	0.23
Night sweats	11 (40.7%)	16 (59.3%)	0.29
Anorexia	17 (42.5%)	23 (57.5%)	0.26
Weight loss	30 (51.7%)	28 (48.3%)	0.69
Dyspnea	3 (27.3%)	8 (72.7%)	0.12
Lower limb edema	15 (46.9%)	17 (53.1%)	0.67

Although the treating clinician requested an ascites cell count in all cases, some subjects had financial or logistical issues, and only 54 out of 103 subjects had this analysis performed. Among those patients who had ascites cell count performed, we found no association between ascites white blood count and ADA level. Among the 23 subjects with an elevated ascites WBC, 17 of them (73.9%) had a high ADA level and 6 (26.1%) had a low ADA level, but this difference was not statistically significant.

Due to financial and logistic challenges, some subjects did not do serum ascites albumin tested in order to calculate the Serum Ascites Albumin Gradient (SAAG). The correlation between SAAG and ADA value was statistically significant (*p* = 0. 039).

## Discussion

In this study we found significant deficits in the tuberculosis treatment of patients with ascites. First, there was a large amount of undertreatment: three out of four subjects with a presumptive diagnosis of peritoneal TB were not given treatment for their disease. Second, there was also significant overtreatment: among the patients who did get anti-TB medications, almost one-fourth did not actually have tuberculosis, and were being exposed to side effects for no reason.

Confirmation of peritoneal tuberculosis is difficult because it presents similarly to other intra-abdominal diseases, and confirmation can require a long time due to the slow growth of mycobacterial cultures [4, 5]. In addition, sensitivity of ascites fluid culture or laparoscopic peritoneum tissue biopsy varies between 43 and 83% depending on the quantity of ascites cultured or quality of tissue biopsied and method utilized.^10.^ For this reason, the use of adenosine deaminase as an added diagnostic tool would be very helpful to avoid undertreatment and overtreatment in low-resource settings such as Rwanda.

In our study, the majority of subjects were under age 55. This is similar to the study done by Muhammad A. Saleh and al where the mean age of the study population was 51 years. Concerning gender, our findings are similar to those found by Kang SJ, Kim JW et al. with sex ratio (M: F) 1: 0.9 and 1:0.6 respectively [9, 10].

We found a prevalence of peritoneal tuberculosis, based on elevated ascites fluid adenosine deaminase level, that is higher than what L.J. Burges et al. and Arnoldo Riquelme et al. found in their respective studies done in 2001 in South Africa and 2006 in Chile. Prevalence in those studies was 10 and 19%, respectively. This difference may be explained by the fact that in their studies they compared ADA level in ascites fluid with peritoneal fluid culture or tissue biopsy [4, 11].

We found no significant association between subjects’ clinical characteristics and their ADA values. This supports our existing understanding of peritoneal tuberculosis, which is that clinicians are unable to differentiate between tuberculous and non-tuberculous ascites by clinical features only. Our data are further confirmation of prior studies suggesting that the ADA test is an important diagnostic tool, because it can change the decision of the clinician about whether to give anti-TB medications or not.

Just under half of the subjects with cirrhosis (39.5%) were found to have ADA level **≥** 39 UI**.** This number is greater than what Muneef M et al. found in 2001, which was that 13% of subjects with underlying chronic liver disease had peritoneal TB. It is less, however, than what Shaki AO et al. found, which was that 62% of patients with peritoneal TB had chronic liver disease [12–14]. Cirrhotic patients are often not treated for peritoneal tuberculosis in Rwanda, because of the assumption that there is an alternate cause of fluid collection. The fact that more than a third of cirrhotic subjects in our study had high ADA suggests that this clinical practice may be misguided. Patients with cirrhosis have an increased susceptibility to developing peritoneal TB [4]. This co-morbidity of cirrhosis and peritoneal TB may explain the high level of ADA in our study population. Further studies should be done to determine what proportions of these cirrhotic patients have peritoneal tuberculosis and should be given anti-TB medications.

There are several limitations to this study. First, due to lack of resources we were unable to perform mycobacterial culture or peritoneal biopsy. Many previous investigations, however, have shown that ADA level is highly correlated with culture and biopsy. A meta-analysis of 12 such studies confirmed that when a cut-off value of 39 is used, the sensitivity of ADA is 100% and the specificity is 97% when compared against culture and biopsy. For this reason, we are confident that our findings are clinically relevant, despite the absence of the gold standard.

Another limitation to this study is the sample size, and the fact that we recruited subjects at tertiary referral hospitals. Many Rwandans first present to a rural health clinic or a Regional District Hospital before arriving at a tertiary center, and our conclusions should not be generalized to this larger population without further research.

We found a statistically significant inverse association between ADA level and SAAG. This finding, however, was based on only 60 of the 103 subjects enrolled in our study. It may be that the group of subjects who had the resources to obtain ascites and serum albumin testing were different than the remaining 43 subjects who did not have this testing done. Our findings regarding ADA level and SAAG should be interpreted cautiously.

Despite these limitations, the fact that 3 in 4 subjects with presumed peritoneal TB did not receive treatment for their disease is a striking finding. Equally concerning is the finding that 1 in 4 subjects who got TB treatment likely did not have tuberculosis. Even if the true number of Rwandans who are being undertreated and overtreated for tuberculosis is less than in our study, these findings are very concerning, and should be followed up with a larger study to confirm or disprove them.

## Conclusion

In our findings, majority of patients with peritoneal TB are not getting TB treatments, 25% of patients are taking unnecessary TB treatment and samples with low albumin gradient ascites were more likely to have high ADA > 39 IU/l (*P* = 0.039). Confirmation of peritoneal TB is difficult and requires a long time due to the slow growth of mycobacterial culture and sensitivity of ascites fluid culture or laparoscopic peritoneum tissue biopsy varies between 43 and 83%. Adding ADA as diagnostic tools available to clinicians could help to avoid under and over treatments of peritoneal TB.

## Data Availability

The datasets used and analysed during the current study available from the corresponding author on reasonable request.

## Abbreviations

ADA: Adenosine deaminase
AKI: Acute kidney injury
CKD: Chronic kidney disease
CHF: Congestive heart failure
HCV: Hepatitis C virus
HBV: Hepatitis B virus
IRB: Institutional Review Board
PTB: Peritoneal tuberculosis
TB: Tuberculosis
SAAG: Serum Ascites Albumin Gradient
CMHS: College of Medicine and health sciences
KUTH: Kigali university teaching Hospital
BUTH: Butare university teaching Hospital
RMH: Rwanda Military Hospital
HIV: Human Immunodeficiency Virus
WHO: World Health Organization
